# Audit of Completion of Risk Assessment on Admission & Discharge - Department of Psychiatry, Gulab Devi Teaching Hospital, Lahore

**DOI:** 10.1192/bjo.2026.11840

**Published:** 2026-06-30

**Authors:** Syeda Areeba Siraj, Mudassar Ijaz, Muhammad Mansoor Ali, Summan Jannat, Syed Muhammad Haseeb Siraj

**Affiliations:** 1 Al Aleem Medical Collge / Gulab Devi Teaching Hospital, Lahore, Pakistan; 2 Avicenna Hospital, Lahore, Pakistan; 3 York and Scarborough Teaching Hospital, York, United Kingdom; 4 Nishtar Hospital, Multan, Pakistan

## Abstract

**Aims:**

To assess compliance with recommended standards for completion and documentation of risk assessment on admission and discharge in the psychiatry inpatient unit, and to evaluate the effect of targeted interventions on improving patient safety and continuity of care.

ET AL AUTHORS:

6. Dr. Ali Abbas Zahid - Internal Medicine PGR- aliabbaszahid004@gmail.com- Aleem Medical Collge / Gulab Devi Teaching Hospital, Lahore, Pakistan

7. Dr. Muhammad Zubair - General Practitioner-*muhammadzubair1103@gmail.com*

**Methods:**

A closed-loop quality improvement audit with a repeated cross-sectional design was conducted over two months. Twenty-five consecutive patients were reviewed in each cycle (total n=50). Baseline data were collected retrospectively from clinical records, followed by implementation of interventions including staff education, display of risk-assessment checklists in the ward, and reminders during ward rounds to complete admission and discharge risk documentation. A prospective re-audit was then performed using identical standards. Criteria assessed included completion of risk assessment within 24 hours of admission, documentation in clinical notes, coverage of risk domains (risk to self, risk to others, risk from others, risk of absconding), documentation of a risk management plan, and review and update of risk assessment prior to discharge.

**Results:**

Admission risk assessment within 24 hours improved from 64% to 92% (absolute improvement +28%). Documentation in clinical notes increased from 84% to 96% (+12%). Assessment of risk to self improved from 84% to 96% (+12%), risk to others from 80% to 92% (+12%), risk from others from 68% to 88% (+20%), and risk of absconding from 80% to 92% (+12%). Documentation of a risk management plan increased from 72% to 88% (+16%).

Discharge-related safety showed the greatest improvement. Review of risk assessment prior to discharge improved from 40% to 84% (+44%), and updating of risk assessment before discharge improved from 40% to 80% (+40%). Overall, post-intervention compliance was high across all admission and discharge standards, with the largest absolute gains seen in safeguarding assessments and discharge safety processes.

**Conclusion:**

This closed-loop audit demonstrates that simple, low-cost interventions led to substantial absolute improvements (12–44 percentage points) in multiple components of risk assessment, particularly in safeguarding domains and discharge risk review, which are critical for preventing post-discharge adverse events. Although post-intervention compliance was high, gaps remain in achieving universal documentation of risk management plans and discharge updates. Embedding standardized admission and discharge proformas and continued staff reinforcement are recommended to sustain and further improve patient safety practices.

